# Sensitivity Modeling Study for an Ozone Occurrence during the 1996 Paso Del Norte Ozone Campaign

**DOI:** 10.3390/ijerph5040181

**Published:** 2008-11-26

**Authors:** Duanjun Lu, Remata S. Reddy, Rosa Fitzgerald, William R. Stockwell, Quinton L. Williams, Paul B. Tchounwou

**Affiliations:** 1 Department of Physics, Atmos. Sci. & Geoscience, College of Science, Engineering and Technology, Jackson State University, 1400 Lynch Street, Jackson, Mississippi, USA; 2 Physics Department, University of Texas at El Paso, 500 W. University Avenue El Paso, Texas 79968, USA; 3 Howard University Program of Atmospheric Sciences, Howard University, Washington DC 20059, USA

**Keywords:** Air quality modeling, Sensitivity study, High ozone event, CMAQ, SMOKE, WRF

## Abstract

Surface ozone pollution has been a persistent environmental problem in the US and Europe as well as the developing countries. A key prerequisite to find effective alternatives to meeting an ozone air quality standard is to understand the importance of local anthropogenic emissions, the significance of biogenic emissions, and the contribution of long-range transport. In this study, an air quality modeling system that includes chemistry and transport, CMAQ, an emission processing model, SMOKE, and a mesoscale numerical meteorological model, WRF, has been applied to investigate an ozone event occurring during the period of the 1996 Paso del Norte Ozone Campaign. The results show that the modeling system exhibits the capability to simulate this high ozone occurrence by providing a comparable temporal variation of surface ozone concentration at one station and to capture the spatial evolution of the event. Several sensitivity tests were also conducted to identify the contributions to high surface ozone concentration from eight VOC subspecies, biogenic VOCs, anthropogenic VOCs and long-range transportation of ozone and its precursors. It is found that the reductions of ETH, ISOP, PAR, OLE and FORM help to mitigate the surface ozone concentration, and like anthropogenic VOCs, biogenic VOC plays a nonnegligible role in ozone formation. But for this case, long-range transport of ozone and its precursors appears to produce an insignificant contribution.

## 1. Introduction

Ozone (O_3_) is of concern due to its adverse effects on both human health and the environment. Breathing ozone can trigger a variety of health problems including chest pain, coughing, throat irritation, and congestion. It can worsen bronchitis, emphysema, and asthma. Ground-level ozone also can reduce lung function and inflame the linings of the lungs. Repeated exposure may permanently scar lung tissue. But human exposure to high concentration of ground level ozone continues to bother many areas in the US in spite of the implementation of government-mandated emissions control strategies [29]. This is because the control of ground level ozone is more difficult than for many other primary pollutants because ozone is a secondary pollutant. In case of other primary pollutants, a reduction in emissions results in an approximately proportional reduction of pollutants. However, as a secondary pollutant that is formed from primary pollutants and other chemical species in the atmosphere, ozone does not necessarily respond in a proportional manner to reductions in precursor emissions [11]. Air quality modeling provides a good alternative to study the physical and chemical mechanism of ozone formation because modeling can provide good temporal and spatial resolution for a wide variety of pollutants. For instance, regional air quality models such as the US EPA’s Community Multiscale Air Quality model (CMAQ/Models-3) can be used to generate hourly ambient concentration fields for ozone, PM10, PM2.5, and many other pollutants, which allows researchers to study the relationship between pollution and health outcomes for times, locations, and pollutants for which monitoring data are not available.

Ozone generation is typically favored in high-pressure, stagnant atmospheric systems at locations that have substantial concentrations of nitrogen oxides (NOx) and volatile organic compounds (VOCs) [16]. Both NOx and VOCs originate from either anthropogenic source sectors (e.g., industrial and transport sectors) or from biogenic sources. Vehicles and gasoline-burning engines are large anthropogenic sources of VOC. VOC emission also comes from consumer products such as paints, insecticides, and cleaners as well as industrial operations, solvent usage, landfills, and waste facilities, petroleum refining, and chemical manufacturing. In addition, vegetation is another major source of VOC emission [1]: about 98% of the estimated total natural VOC emissions are from vegetation [15]. Many previous ozone control measures rely on reducing emissions of VOCs. VOC controls are traditionally based on the total mass of organic compounds, not taking into account the unique characteristics of each individual subspecies [16]. However, individual VOCs behave differently in the atmosphere and have differing ozone formation potentials. In accordance with the study of Cardelino and Chameides [5], the contribution of biogenic emission has previously been underestimated in air quality modeling. On the other hand, long-range transport of pollution has been reported to give an essential contribution to local ozone concentration [27].

**Figure 1:**
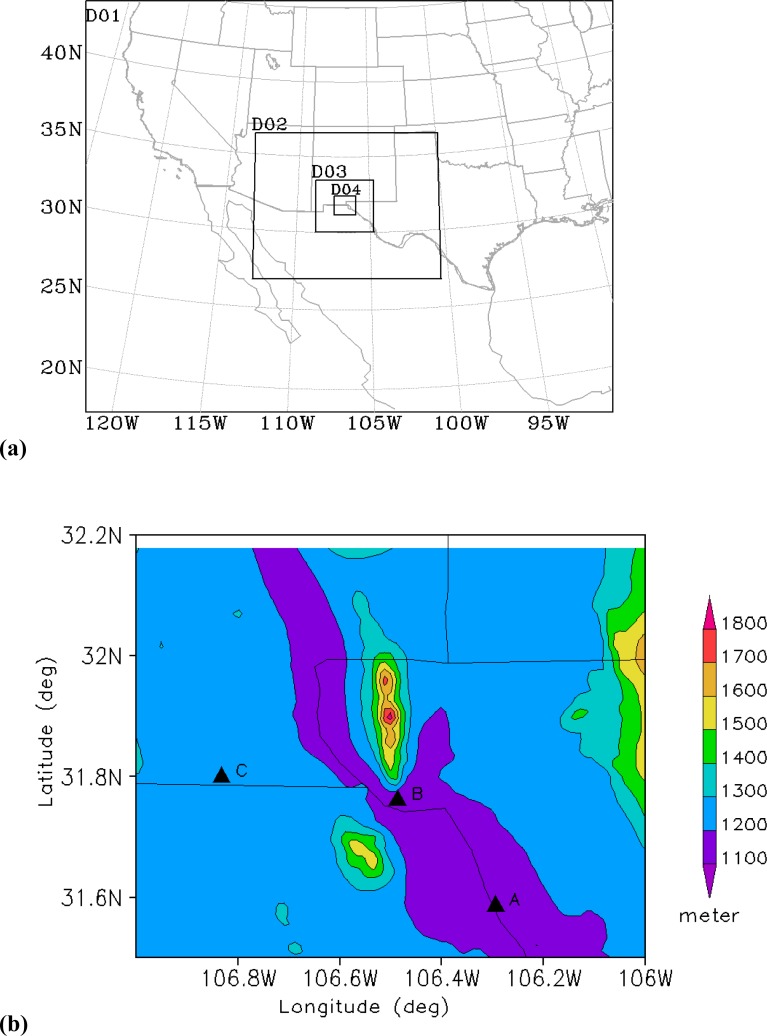
**(a)** Nested domains used for WRF, SMOKE and CMAQ models. D01, D02, D03 and D04 are the outer, middle and fine domains with the grid distances of 36 km, 12 km, 4 km and 1 km respectively. **(b)** Fine domain (1km) topography (meters). Three points are selected, and Point B is the location of downtown El Paso. The northern high topography area is the Franklin Mountains and its counterpart to the south is the Juarez Mountains. The Rio Grande River valley runs between these two mountains through El Paso.

In this work, an air quality modeling system was applied to a historic case occurring in the 1996 Paso del Norte Ozone Campaign (see Fig. 1) to study the relationships between ozone and its natural and anthropogenic precursors. Some studies have been conducted to investigate the 1996 Paso del Norte Ozone Campaign in order to develop an understanding of the chemical and physical processes which influence high ozone concentrations in the Paso del Norte study area [2, 9, 12, 13, 23, 31]. Most of these studies were diagnostic, and modeled the case by adapting an idealized profile [2]. According to the studies of Trainer *et al*. [35] and Cardelino and Chameides [4], three key ingredients are needed for understanding ozone precursor relationships: the relative concentrations of VOC and NOx, the importance of natural VOC relative to anthropogenic VOC in the atmosphere, and the significance of long range transport versus local emissions of ozone precursors. It was found that ozone events are a product of both long distance transport of pollutants and in-situ photochemical production. With only an observational diagnosis, we cannot reach a conclusion as to the relative impacts of long-range transport of ozone and its precursors versus local emissions on the ozone events. Lee *et al*. [21] found that using surface data assimilation in a meteorological model results in a clear improvement for ozone concentration simulation over either mountainous or flat areas. For this reason, a modeling study with high resolution and a data assimilation scheme is needed. Here we report the results of a modeling study using a next generation meteorological model, such as Weather Research and Forecast (WRF) model [32], to investigate a high ozone concentration case that occurred in 1996 in the Paso del Norte area. We do so by coupling the Community Multi-Scale Air Quality (CMAQ) model [3]. The focus of this modeling study is on (1) investigating the relative impact of long-range transport of ozone and precursors versus local emissions on high ozone occurrences, (2) studying the relative contribution of biogenic VOC emissions and anthropogenic VOC emissions to local ozone production during ozone events, and (3) identifying the specific contribution from each VOC subspecies toward ozone formation. In this study, CB-IV was used as a chemical mechanism [1] in CMAQ, where there are 10 subspecies including ETH (ethene), ISOP (isoprene), PAR (paraffin group, molecules containing single carbon bond groups), OLE (olefin group, molecules containing double carbon bond groups), TOL (toluene group, 7-carbon rings), XYL (xylene group, 8-carbon rings), ALD (aldehyde group), FORM (formaldehyde), NR (non reactive VOC group), and TERP (terpene group).

## 2. Case selected and model configuration

### 2.1. Selected case

The case analyzed here, the 13 August 1996 ozone episode, was selected from a major field study, the Paso del Norte Ozone Study, which was conducted during the summer of 1996 for providing sufficient data to support photochemical ozone air quality modeling and to develop an understanding of the chemical and physical processes which influence high ozone concentrations. The National Ambient Air Quality Standard (NAAQS) was violated on 13 August 1996 in El Paso/Juarez area [23]. For instance, the Chamizal (31.7681N, 106.4542W) and Campbel (31.7625N, 106.4869W) stations in El Paso reported maximum hourly averages of 137 and 136 ppbV, respectively, relatively early in the day at 11.30 h MST. The 20/30 Club station (31.74N, 106.47W) in Juarez just to the south of Chamizal had a very early peak of 126 ppbV at 09.30 h MST. Monitoring stations in the surrounding rural areas had much lower values during the morning hours. Later in the day, the UTEP site (31.7683N, 106.5006W) on the western side of El Paso observed a 124 ppbV value at 12.30 h MST. The Desert View (31.7961N, 106.5839) and Sunland Park (31.7958N, 106.5575W) stations to the west and downwind of El Paso had maximum ozone values of approximately 110 ppbV occurring much later in the day at 15.30 h MST. It was also observed that an ozone (ozone precursors) cloud forming over the urban core of Juarez and El Paso was advected to the northwest by the prevailing southeasterly winds on that day.

### 2.2. Frameworks of models

The air quality modeling system used in this study is composed of three main components: a meteorology model (WRF), a chemistry and transport processor (CMAQ) and an emissions model (SMOKE). In addition, an interface processor between the meteorology and chemistry (MCIP) was also used to generate necessary 2D and 3D meteorological input for both CMAQ and SMOKE. Detailed information about these models is beyond the scope of this paper, but a brief description is given in the following.

#### 2.2.1. WRF

The version used in this study is the Advanced Research WRF (ARW) core 2.2 [32]. WRF is a multi-institutional effort to develop an advanced mesoscale forecast model and data assimilation system that will advance both the understanding of mesoscale prediction systems and the quality of their predictions, and to promote closer ties between the research and operational forecasting communities. The parameterizations employed by WRF consist of a level-1.5 order turbulence kinetic energy closure scheme, 5 layers in the land surface model, Ferrier *et al*. [10] microphysics, RRTM long wave radiation scheme [25], simple short wave radiation [8], YSU PBL scheme [26] and the modified version of the Kain-Fritsch [20] convective scheme.

#### 2.2.2. CMAQ

The chemical transport model used in this study is the Community Multiscale Air Quality Model (CMAQ) developed by the US EPA [3]. CMAQ is a comprehensive, three-dimensional, multiscale, Eulerian-based, atmospheric chemistry, transport and deposition model for multiple air pollutants including tropospheric ozone, acid deposition, particulate matter, visibility and air toxins. It is formulated using a generalized coordinate system that facilitates its linkage with different meteorological models. CMAQ serves as a flexible and comprehensive modeling tool in various applications such as urban and regional air quality evaluations, formulating state implementation plans for non-attainment areas, and investigating atmospheric processes [22]. Table 1 lists the major physical and chemical processes used in this study.

#### 2.2.3. SMOKE

The emission inventory model used in this study is the Sparse Matrix Operator Kernel Emissions (SMOKE) Modeling System developed by Carolina Environmental Program at the University of North Carolina at Chapel Hill [17–18]. The main goal of SMOKE is to convert the source-level emissions (county total emissions) reported on a yearly basis to spatially resolved, hourly emissions, with detailed speciation information. By ingesting inputs on landuse, meteorological fields, and emission inventory data, SMOKE can generate the speciated, gridded emission inventory inputs for various air quality models, including CMAQ. SMOKE is able to deal with point, area, biogenic, mobile and point emission sources: point source emissions are gridded according to the physical location of emitting facilities, area and mobile source emissions are gridded according to the spatial allocation factors, and biogenic emissions are calculated using Biogenic Emission Inventory System Version (BEIS) version 3.13 along with meteorological data such as surface solar irradiation and temperature. A total of 39 gaseous and PM species were included in the emission processing.

#### 2.2.4. MCIP

There is an interface processor in the modeling system to incorporate the outputs from the meteorology model and to prepare the required information on the initial and boundary conditions and photolysis rates to both the air quality and emission components: the Meteorology-Chemistry Interface Processor (MCIP). Since most meteorological models, including WRF, are not built for air quality modeling purposes, the outputs cannot be implemented directly to air quality models. The MCIP program translates and processes model output from the WRF model for the CMAQ and the SMOKE. The MCIP handles data format transformation, conversions of units of parameters, extraction of data for appropriate window domains, collapsing of meteorological profile data if coarse vertical resolution data are required, computation of cloud parameters, surface and PBL parameters, and species-specific dry deposition velocities.

### 2.3. Input Data

The data used as input to the WRF model as initialization and lateral boundary conditions is obtained from the NCEP/NCAR Reanalysis at 6 h intervals. This is a global dataset in grib format, with a resolution of 2.5 x 2.5 degrees. The emission inventory data used in this study are EPA’s NEI99 (final version 2), available from ftp://ftp.epa.gov/EmisInventory. The inventoried emissions in NEI99 include nitrogen oxides (NOx), sulfur dioxide (SO2), volatile organic compounds (VOCs), carbon monoxide (CO), ammonia (NH3), and particulate matter (PM) for point, area, nonroad and on-road mobile emissions. Since the simulation is located at the region that covers the US and Mexico, the latest released Mexico emission dataset (Mexico NEI99. http://www.epa.gov/ttn/chief/net/mexico.html), which include six northern border states of Mexico, has also been obtained for to supplement NEI99.

### 2.4. Design of numerical simulations

The WRF model run used a four-level nested one-way domain scheme centered at the location of El Paso, TX (31.70N, 106.40W) with the spatial resolution of 36km, 12km, 4km and 1km for the outer, middle and inner domains, respectively (Fig. 1). The inner most domain has the coverage of 140km × 140km which includes the whole area of interest. The high resolution will help the meteorological model to resolve the unique weather phenomena occurring over a complex area such as El Paso [2], where mountainous topography produces regional circulation that results in wind in the mountains and valleys, which consequently affects the local pollutant transportation and dispersion. The vertical layers of WRF contain 35 sigma levels with 15 layers within the planetary boundary layer (PBL) (less than 1500 m), in which the lowest sigma level is at a height of 12 meters. During the processing of MCIP, vertical layers were collapsed into 26 levels to alleviate the computational costs while all layers within the PBL have been kept to maintain the resolution at the elevation where emission and chemical reactions of pollutants occur. Similar practices are standard in the literature on air quality modeling [7, 22, 24]. In addition, the analysis nudging scheme of Four Dimensional Data Assimilation (FDDA) (Stauffer and Seaman 1994) has been applied to the inputs of the meteorological model for the first 6 hour simulation in all domains in order to mitigate the meteorological uncertainties. In WRF modeling, the nudging technique has been implemented for winds, temperature and water vapor. All models were run for a 4 day period starting from 1200UTC August 10, 1996. The CMAQ model was launched by applying cold starting at 1200UTC August 10, 1996. Since the idealized profiles of chemical species were ingested into the CMAQ initially, the first three-day simulation of the CMAQ was used to allow the model to spin up. This common method for cold starting that has been used in many researches during past. Although the results are available for all domains on all days, the focus of the analysis in this study is on the finest domain on last day when the ozone standard was not attained.

### 2.5. Sensitivity study

A base-case was simulated using the above mentioned specification. After that, a number of sensitivity studies were carried out to compare with the results from different emission scenarios, where the original emission from SMOKE was perturbed by the factor of 50% for each CB-IV VOC subspecies in CMAQ including ETH, ISOP, PAR, OLE, TOL, XYL, ALD, NR and FORM. TERP was not included in the sensitivity test because of no availability from SMOKE. Another simulation was then conducted without ozone and its precursor at four lateral boundaries to test the impact of long-range transport. Additional, simulations were carried out to test the relative contributions of biogenic VOC and anthropogenic VOC to local ozone production.

## 3. Results and discussion

### 3.1. Evaluation of meteorological parameters

**Figure 2:**
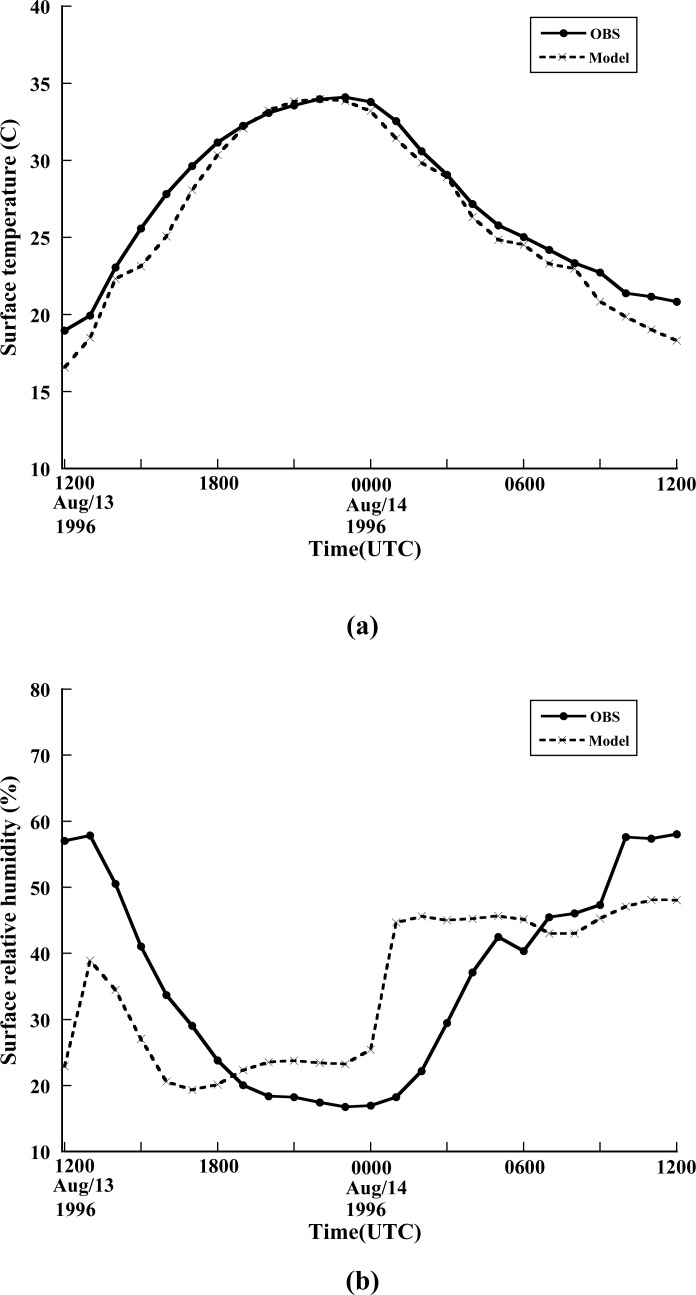

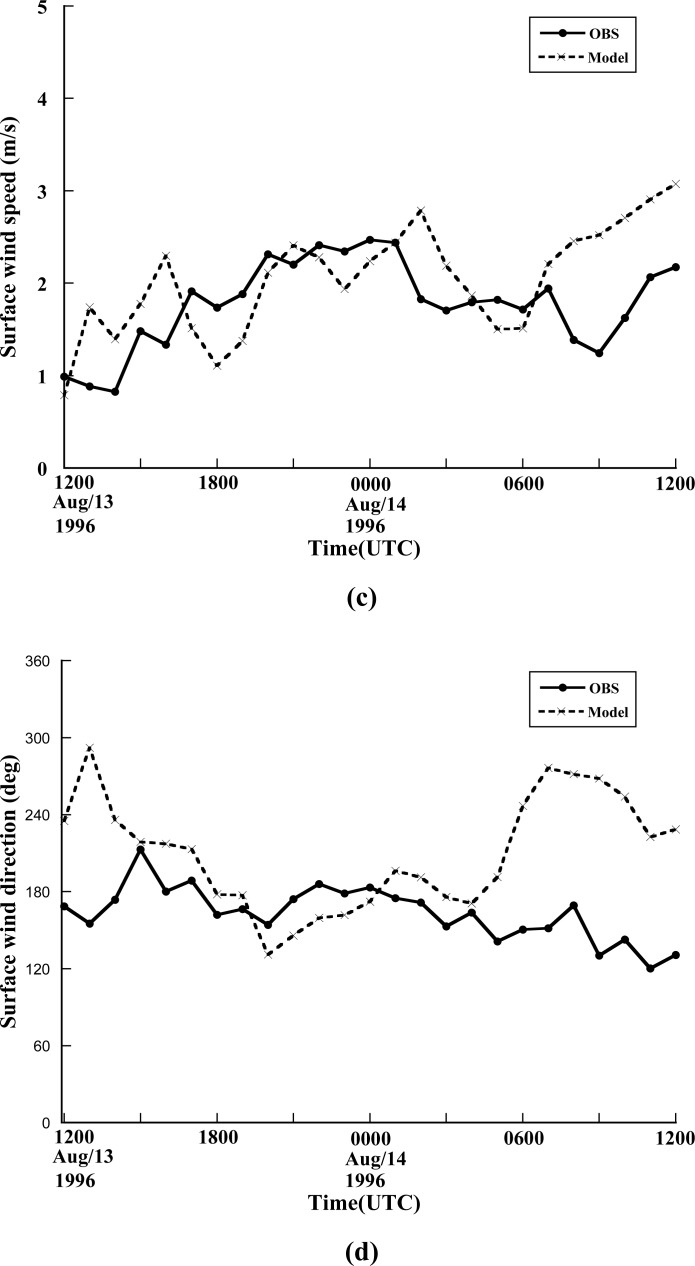
The time series comparison of hourly observed and predicted domain-averaged surface (a) temperature, (b) relative humidity, (c) wind speed, and (d) wind direction during the period from 1200Z 13 August to 1200Z 14 August 1996. The average is performed in fine domain (1km resolution).

The ozone formation depends on both meteorological and chemical conditions. It is necessary to evaluate meteorological simulation before studying air quality prediction. The meteorological variables such as temperature, wind, pressure and humidity play an important role in ozone initiation, development and depletion. The extensive investigation for meteorological evaluation is beyond to the scope of this paper. A brief description of meteorological simulation is documented following. Various surface and upper-level air quality measurements were taken during the 1996 Paso del Norte Ozone Campaign [28]. Figs 2(a)–(d) show the time series of hourly observed and predicted surface temperature, surface relative humidity and surface wind speed. The observed data are areal values averaged by 21 surface stations [28] within the fine domain (1 km grid distance) area. The corresponding modeled data are domain-average values. The observed surface temperature and relative humidity are at 2 meter height while surface winds are 10 meter above the ground. The simulated surface variables are located at the midpoint of first layer of CMAQ (12 meter above ground). It is found that the WRF model well reproduced for surface temperature. The diurnal variation of the surface temperature was clearly captured. The normalized bias [36] of the simulated surface temperature is −0.98°C and root-mean-square error is 1.34. The simulated surface temperature agreed very well with the observed during the period of local afternoon when temperature reached its highest value. For surface relative humidity, wind speed and wind direction, the model simulations were also reasonably good compared to the observed. The relative dry period occurring during afternoon was captured by the model. The model produced normalized biases at the amounts of −1.27%, 0.26 m/s and 46° for surface relative humidity, wind speed and wind direction respectively. As addressed in Section 2.4, FDDA scheme was used in this study to improve the meteorological simulation. It is found that the application of FDDA decreased the normalized bias of surface temperature from −2.25 °C to −0.98°C, of surface relative humidity from −1.42% to −1.27%, of surface wind speed from 0.73 m/s to 0.26 m/s, and of surface wind direction from 51°C to 46°C.

### 3.2. Base-case simulation

**Figure 3:**
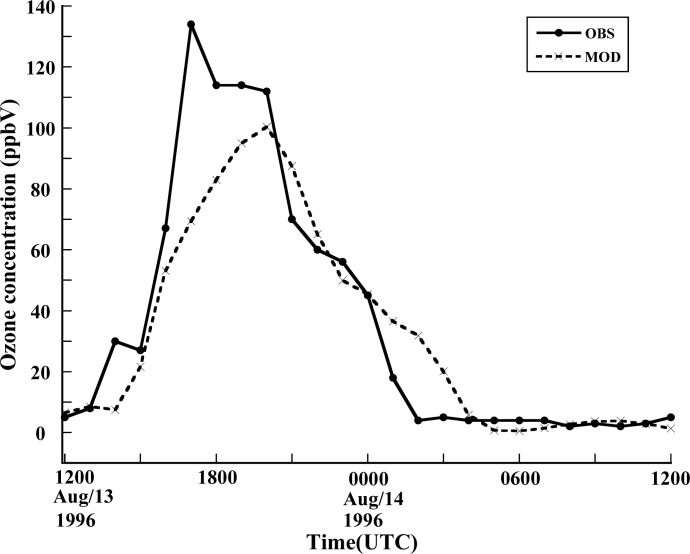
Surface ozone concentration comparison between model and observation at the location of downtown El Paso during the period from 1200Z 13 August to 1200Z 14 August 1996.

**Figure 4:**
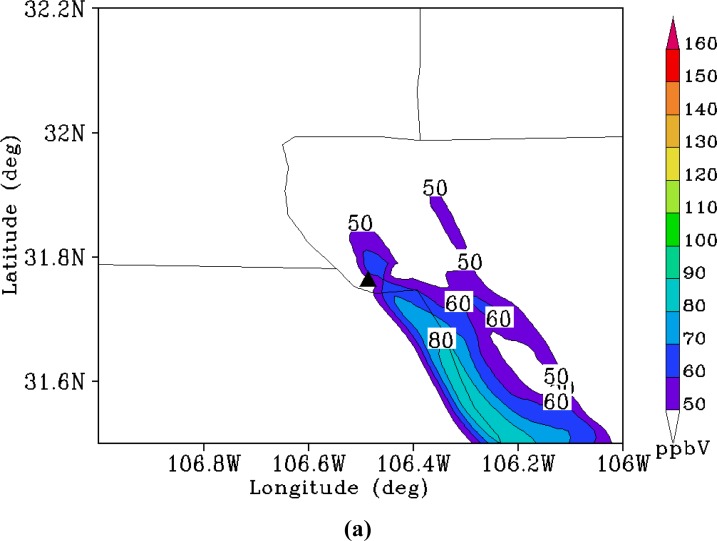

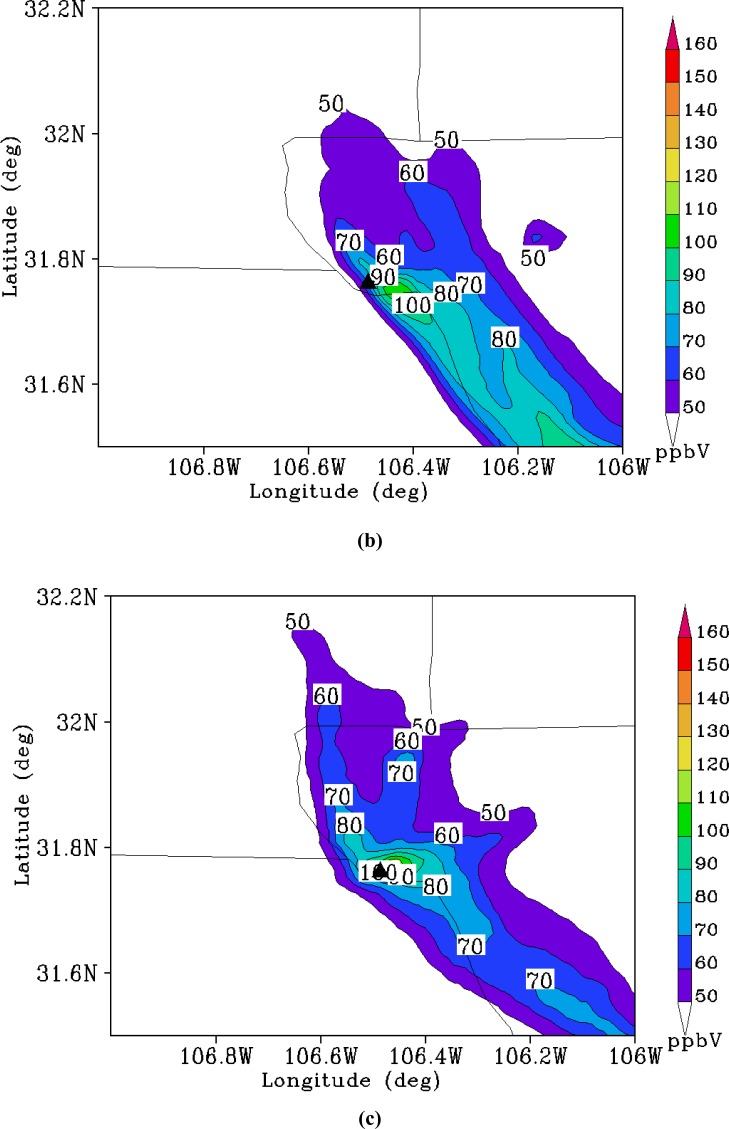

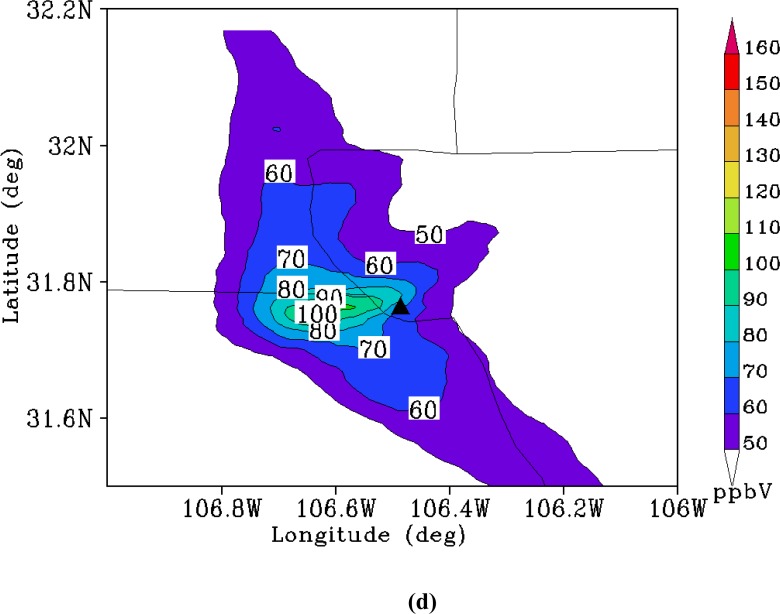
Simulated surface ozone concentration (ppbV) in the fine domain (1km spatial resolution) at (a) 1000 MST, (b) 1200 MST, (c) 1400 MST, and (d) 1600 MST. Black triangle denotes the location of downtown El Paso.

**Figure 5:**
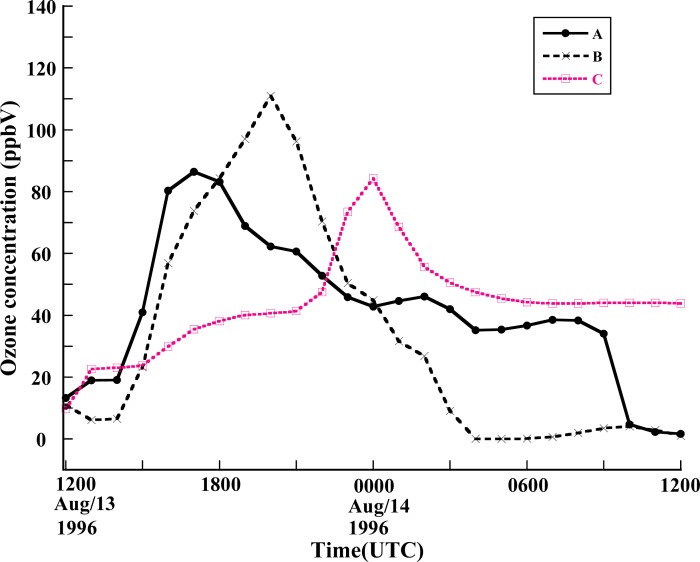
Comparison of surface ozone concentrations at Point A, B and C in Figure 1b.

The base case was selected to cover the period from 12UTC 13 August to 12UTC 14 August. Figure 3 shows the comparison of 1h surface ozone concentration at the site of El Paso from observed and simulated data. The observation of ozone was obtained from one station in downtown El Paso during the period of the 1996 Paso del Norte Ozone Campaign [28]. It is found that there is a reasonable agreement between the model simulation and the observations in general. A clear diurnal variation of surface ozone concentration has been simulated. The simulation yielded a peak value of 101 ppbV while the observed peak value was 134ppbV, which indicates that the CMAQ model underpredicted the peak value of the surface ozone concentration compared to the observed value. Also, the surface concentration of ozone peaked 3 hours later in the simulation then in the observed data. The downtown El Paso station reported a maximum 1h ozone concentration of 134ppbV at 1700Z 13 August (1100 MST), while the model gave the maximum concentration as occurring at 2000Z (1400 MST). This delay may come from the simulation errors in the surface temperature, wind speed or humidity as well as uncertainties in emission distribution and intensity. Further detailed investigation is needed to understand this issue. Roberts *et al*. [28] reported that that an ozone cloud formed over the urban areas of Juarez and El Paso and flowed northwest due to the prevailing southeasterly winds. The CMAQ model simulation captured the evolution of this pattern (Figure 4). Figure 4a shows the model simulation of surface ozone concentration at 1000 MST. It is found that high ozone concentration areas are located along the Rio Grande River Valley, where the US and Mexican border is located. The maximum ozone value reaches up to 80 ppbV oriented southeast-to-northwest. At 1200 MST (Fig. 4b), the maximum center of the simulated surface ozone concentration moves northwestward to the southeastern part of El Paso city with an intensity of 101 ppbV. During the next 2 hour period from 1200 MST to 1400 MST, the maximum center barely moves forward. But the surface ozone concentration intensifies to 112 ppbV at 1400 MST (Fig. 4c). After this period, the high surface ozone area moves westward along the border. At 1600 MST, the center is located approximately 10km west of El Paso, with an intensity of 100 ppbV. This unique track of high ozone is very much associated with the local topography [28], especially under the ozone favoring conditions associated with calm weather. As seen in Figure 1b, the Paso del Norte study area is located at the western corner of Texas that adjoins New Mexico and Chihuahua, Mexico. Most of this area is desert, with agriculture along the Rio Grande River. The main geographical features are the Franklin Mountains, which run north-to-south and end abruptly just north of downtown El Paso; the Juarez Mountains, which lie to the west of Ciudad Juarez; and the Rio Grande River valley that divides the Franklin and Juarez Mountains and runs generally northwest-to-southeast through the domain. During summer, these geographical features strongly influence the local surface winds in the presence of the large-scale high pressure systems that predominate in this area. The typical summer day begins with drainage flow down the Franklin and Juarez Mountains and the Rio Grande River Valley [28]. This flow results in light northwesterly winds in the area. As the morning sun warms the east and south sides of the Franklin and Juarez Mountains, the drainage flow weakens. As heating continues throughout the day, the winds reverse direction and become upslope winds from the south and east. Consequently, surface ozone is advected northwestward by the prevailing winds. But the Franklin mountains eventually block the further movement of the winds and deflect them westward, which results in the spatial pattern of the simulated surface ozone distribution. Figure 5 shows the temporal variation of the surface ozone concentration at three points in Figure 1b. Three-hour delays separate the occurrences of peak ozone concentration at points A and B, and between points B and point C.

### 3.3. VOC subspecies contributions

In terms of the CB-IV chemical mechanism [3] in the CMAQ, the VOC can be appointed into 10 subspecies, based on their molecular structural. The subspecies are Ethene (ETH), Isoprene (ISOP), Paraffin carbon bond (PAR), Olefinic carbon bond (OLE), 7 carbon aromatics (TOL), 8 carbon aromatics (XYL), Acetaldehyde and higher aldehydes (ALD2), Methyl glyoxal (MGLY) and Cresol and higher molecular weight phenols (CRES). Since MGLY and CRES are not available from the SMOKE, eight VOC subspecies, ETH, ISOP, PAR, OLE, TOL, XYL, and ALD2, were perturbed by 50% to investigate the influence of each subspecies on ozone formation. Isoprene has a short-life. Toluene and xylene are tracer species produced by both vehicle exhaust and solvent sources. In contrast, ethene is a tracer species for vehicle exhaust. The NR perturbation had no effect on the ozone concentration. Therefore, the contribution of NR is not displayed in following figures. Figure 5a shows the change in the peak ozone concentration in downtown El Paso when each of the VOC subspecies is reduced, one at a time, by a factor of 50%. It is found that decreasing the abundance of a VOC subspecies may either increase or decrease the peak ozone concentration. Decreasing the amounts of ETH, ISOP, PAR, OLE or FORM decreased the peak ozone concentration, while decreasing in TOL, XYL or ALD2 could increase the peak value of surface ozone concentration. TOL gives the largest contribution to peak ozone whereas the XYL group plays the least role in the formation of ozone. Note that each VOC subspecies had a different fraction of mixing ratio prior to the perturbation. To show the contribution for ozone concentration according to per unit mixing ration of subspecies, a new term, ozone potential, has been defined as the ratio between peak ozone difference and the individual subspecies mixing ratios:

Ozone potential=(difference of peak ozone)/VOC subspecies mixing ratio

**Figure 6:**
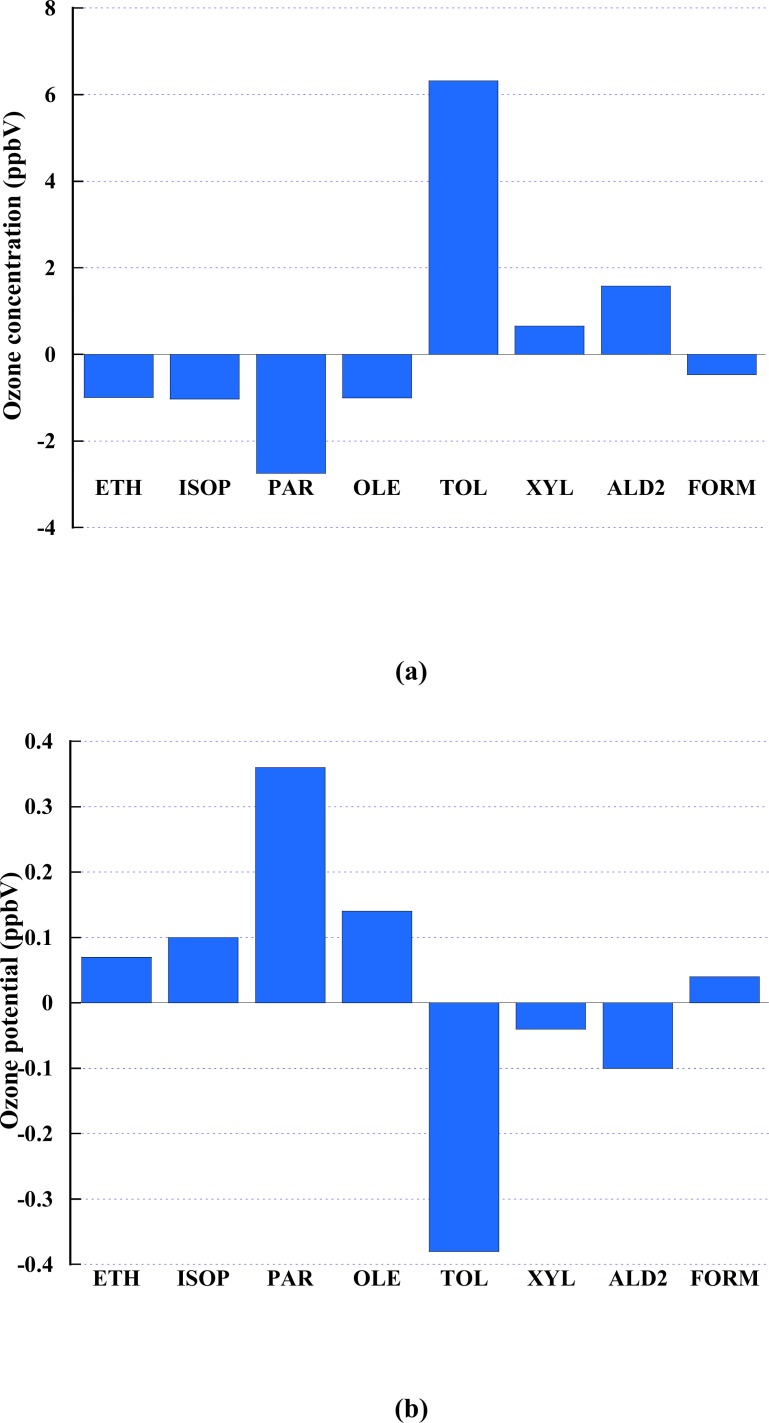
(a) The change in the peak ozone (ppbV) at downtown El Paso after reducing the mixing ratio of each VOC subspecies by a factor of 50%; one subspecies at a time; (b) the ozone potential (ppbV) of the individual VOC subspecies.

This ozone potential tells how much the peak ozone decrease (increase) while VOC decrease (increase) by per unit change of the mixing ratio of VOC subspecies. A positive value indicates that the ozone peak increases (decreases) as the individual VOC’s mixing ratio increases (decreases). A negative value means that the ozone peak decreases (increases) as the individual VOC’s mixing ratio increases (decreases). Figure 6b shows the ozone potential for the individual VOC subspecies. It shows that the PAR group has the strongest positive ozone potential while TOL possesses the most significant negative ozone potential. The ozone potential of the VOC subspecies should be used in a strategic plan for ozone reduction. If certain VOC subspecies has a large positive ozone potential, then decreasing its emission will help reduce the formation of ozone, whereas if it has a negative potential then decreasing its emission is not effective for alleviating ozone concentration, and may even be counterproductive. On the other hand, XYL and FORM subspecies are irrelevant for ozone formation.

### 3.4. Impact of long-range transport

**Figure 7:**
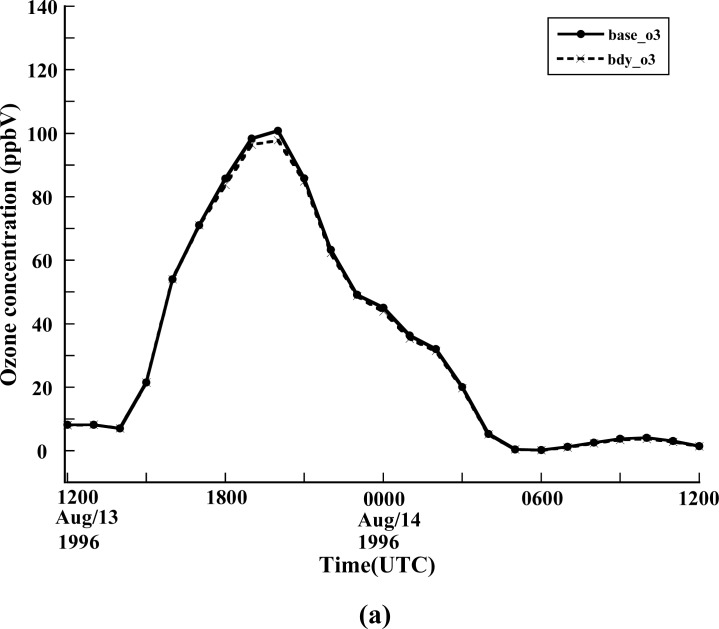

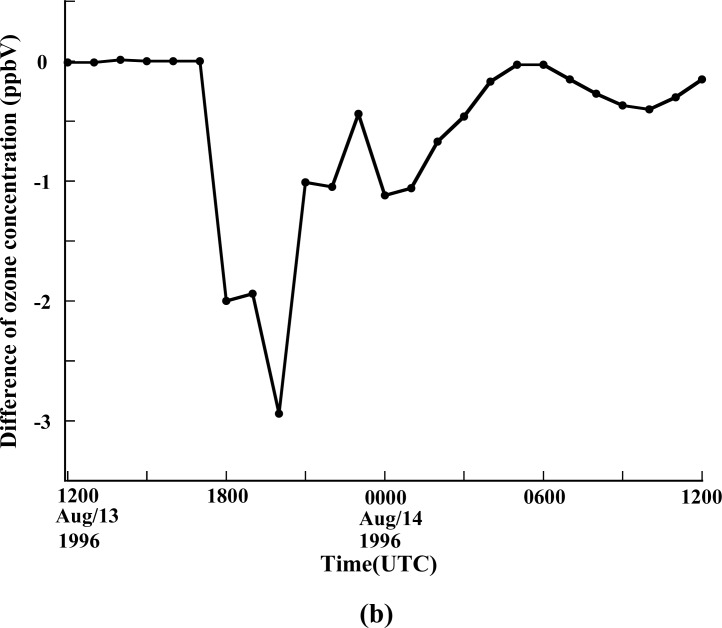
(a) Surface ozone concentration comparison and (b) the difference between base simulation and controlled experiment for lateral boundary input of ozone and its precursors at the location of downtown El Paso during the period from 1200Z 13 August to 1200Z 14 August 1996.

The high ozone events are combinations of long-range transport and local generation. To analyze this, it is necessary to identify and quantify the contributions of long-range transportation of ozone and its precursors, of local anthropogenic emissions, and of local biogenic emissions to ozone occurrences. By analyzing observations and calculating back trajectories for the ozone case of July 1995 in the Baltimore-Washington region, Ryan *et al*. [30] showed the influence of significant regional scale transport of ozone and its precursors on an ozone event. However, the observational data alone do not suffice to distinguish the relative impacts of long-range transport of ozone and its precursors from those of local emissions. In this paper a three dimensional air quality model, CMAQ, has been applied to the case of the 1996 Paso del Norte Ozone Campaign to study the contribution of long-range transportation of ozone and its precursors. Figure 6 shows the comparison and difference of the 1hour surface ozone concentration in downtown El Paso for the base simulation and a lateral boundary control experiment. As Figure 7 indicates, decreasing the lateral boundary input of ozone and its precursors does not significantly impact the surface ozone concentration. The maximum effect of lateral input occurs around local noon when the zone concentration peaks. The largest impact from long-range transport is about 3ppbV (Figure 7b). It is seen that long-range transport of ozone and its precursors has a limited influence for this case, implying that local emission and corresponding photochemical reactions are primarily responsible for the high ozone occurrence.

### 3.5. Contributions of biogenic and anthropogenic VOCs

**Figure 8:**
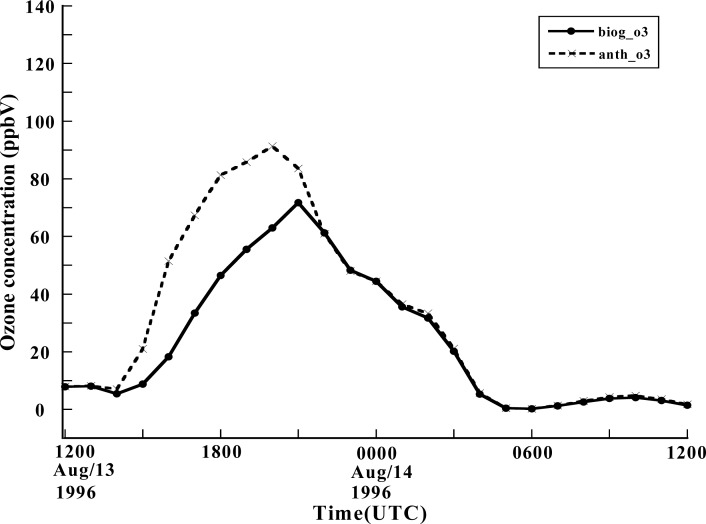
Surface ozone concentration comparison between the output from CMAQ running only with biogenic VOCs (solid line) and only with anthropogeinc VOCs (dashed line) at the station in downtown El Paso.

The VOCs originate from a wide variety of sources, both anthropogenic and biogenic. According to the study by Solomon *et al*. [33], in the US the total amount of reactive VOC emissions from biogenic sources is approximately 1.4 times greater than those from anthropogenic VOC. The present study investigated the relative impact of biogenic VOC emissions versus local anthropogenic VOC emissions on local ozone production. The motivation for this study is that only anthropogenic emissions can be controlled, and several observation-based studies indicate a significant role for biogenic VOC in rural areas as well as in many urban and suburban locations in ozone formation. The emissions from biogenic sources have been reported to be most likely underestimated in past modeling studies, and several analyses of observed measurements have suggested a significant role of biogenic hydrocarbon emissions in ozone formation [4–6]. But the ratio between anthropogenic and biogenic emissions differs greatly from one region to another. According to a study by Guenther *et al*. [15], over 98% of total biogenic VOCs in North America come from vegetation and isoprene, which has a large ozone forming potential, amounting to 35% of the total VOCs emitted from vegetation. To investigate the contributions of anthropogenic and biogenic VOCs to local ozone formation for the case of the 1996 Paso del Norte Ozone Campaign, two sensitivity modeling tests have been conducted: one is a CMAQ model run which excluded anthropogenic VOCs, and another run which excluded biogenic VOCs. Figure 8 shows the 1h ozone concentration in downtown El Paso obtained by running CMAQ with only biogenic VOCs (solid line) and with only anthropogenic VOCs. It is found that both biogenic and anthropogenic VOCs, individually, make a significant contribution for ozone formation. When anthropogenic VOCs are excluded, biogenic VOCs cause the CMAQ model to produce a peak value of 72 ppbV at 1400 MST at the station in downtown El Paso. In contrast, using only anthropogenic VOCs in the model leads to a peak ozone concentration of 91 ppbV at the same location, and about 1.5 hours earlier. Anthropogenic VOCs appear to have more impact on the ozone peak. But we cannot conclude that biogenic VOCs can be ignored in CMAQ. When both types of VOCs were included the peak ozone concentration predicted by the model was not simply a sum of the results from using the two types of VOCs one at a time. This nonlinearity is possibly caused by the complicated chemical reactions occurring among various species in the CMAQ model. In addition, both anthropogenic and biogenic VOCs contributed to the diurnal variation of the ozone concentration.

## 4. Conclusion

A modeling study has been performed for a high ozone occurrence during the 1996 Paso del Norte Ozone Campaign by incorporating a meteorological model (WRF), an emission model (SMOKE) and an air quality chemical and transport model (CMAQ) to investigate the relative contribution of long-range transport versus local emissions, and the relative impact of biogenic VOC emissions versus anthropogenic VOC emissions on the local ozone production. A very fine spatial resolution, 1km, was used in the simulation. The meteorological modeling provided a good agreement of the simulations of surface temperature, relative humidity, wind speed and wind direction against the observations. The simulation results demonstrate the model’s forecast skill in the ozone event. The base case experiment shows a good agreement between the model simulation and the field observations based both on the temporal variation of the surface ozone concentration at one station, and in the spatial evolution of the event. The results also show the local topography impacts the surface ozone transport. To identify the contributions of the various VOC subspecies to a high ozone event, eight subspecies in the CB-IV mechanism in the CMAQ model were investigated by reducing their corresponding concentrations one at a time by a factor of 50%. Decreasing certain VOC subspecies decreased the peak ozone concentration, whereas decreasing other VOC subspecies increased the peak ozone concentration. Decreasing ETH, ISOP, PAR, OLE and FORM would aid control, whereas decreasing TOL, XYL and ALD2 would not decrease the ozone concentration. According to the ozone formation potential, PAR has the largest potential for ozone formation per unit mixing ratio. For the event studied here, the modeling study reveals no significant contribution from the long-range transport of ozone and its precursors. Local emission and its photochemical reactions appear to be the main cause of the high ozone occurrence. The relative contribution of anthropogenic VOC emissions versus biogenic VOC emissions to local ozone production was also investigated. The simulations suggested that biogenic VOC emissions play an important role in local ozone production. These results strengthen the conclusion of Guenther *et al*. [15] that the concentration of biogenic VOC has been underestimated in air quality models. Both anthropogenic and biogenic VOCs have clear influences on local ozone events.

It should be noted that the role of VOCs in ozone formation will be affected by the concentration of NOx. Without considering NOx, but perturbing only the concentration of VOCs, cannot lead us to a definitive conclusion for about ozone formation. No effort has been made in this study to investigate the role of NOx in an ozone event. In the future, several emissions control scenarios for NOx will be tested in a modeling study.

## Figures and Tables

**Table 1:** The major processes used in CMAQ.

**Process**	**Option (settings in CMAQ)**
Horizontal advection:	Piecewise parabolic method (PPM)
Vertical advection:	Yamatino
Horizontal diffusion:	Multiscale diffusion (multiscale)
Vertical diffusion:	Eddy diffusion (eddy)
Photolysis:	Photolytic rate constants (phot)
Plume in grid:	Not invoked (ping_noop)
Gas-phase chemistry:	Carbon Bond IV (CB4) mechanism
Gas-phase chemistry solver:	MEBI/Hertel
Secondary organic aerosol:	SORGAM
Aqueous-phase chemistry:	Regional Acid Deposition Model (RADM)
Aerosol chemistry:	AE4/ISORROPIA
Initial/lateral condition of chemistry:	Idealized profile
